# Initiation into the street, challenges, means of survival and perceived strategies to prevent plights among street children in Addis Ababa, Ethiopia 2019: A phenomenological study design

**DOI:** 10.1371/journal.pone.0272411

**Published:** 2022-08-29

**Authors:** Ayana Chimdessa

**Affiliations:** College of Medicine and Health Sciences, Ambo University, Ambo, Ethiopia; PLOS: Public Library of Science, UNITED KINGDOM

## Abstract

**Background:**

The life and health of street children are becoming a global concern. Push and pull factors i.e. poverty, family death, economic decline, child abuse, financial independence, and peer influence draw children into the street. The street lives by itself pushes them into sex work, and lack of shelter magnificent abuses, where both sexes have the same sleeping quarters.

**Materials and Methods:**

A phenomenological study design was employed from January to March 2019 in Addis Ababa, Ethiopia. Time-Space Sampling (TSS) was used to recruit participants into the study. Focus Group Discussion (FGD) and individual in-depth interview were used to collect data. Data analyzed by using framework analysis software.

**Results:**

About 103 participants took part in the study. Finding shows street children are encountering widespread challenges like social network fragmentation, child trafficking, harassments, and shortage of basic needs. Furthermore, poor design of comprehensive, contextualized strategies and less political value worsening the problems. They are considered as a felon, outlaws, and having a mentality of committing a crime by both law-keeping bodies and society. This situation resulted in fewer acceptances of street children by the community, and less legal protection by law that made them more at risk for denial of social protection. Street children positively perceived strategies like Income Generating Activities (IGAs), shelter, community support, child protection, and access to education, health services, life coaching, and less extent re-integration strategies to address their problems.

**Conclusion:**

The study shows street children are defenseless to harassments and denial of social protection services. Poorly designed policies, strategies targeting them, less political values, and traditional response by government has been subjugated, which made them prone to health and social problems. Therefore, finding might be beneficial to health data scientists, and policymakers; to design and implement policies, and strategic plans in addressing, and preventing their plights.

## Introduction

The childhood period is the time when children are totally being dependent on their families for basic needs and guidance. Children need adequate care, support, protection and guidance services to have healthy growth and development [1]. However, in today’s modern world, due to different pushing and pulling factors dozens of thousands of children are left alone and joined the street, where they are living with the plights of street life [2]. Worldwide, streetism of children continues to grow dramatically. A comparative study shows that a pushing factors like economic 72.6%, family 3.7%, orphaned 2.1%, parent pressure 9.5%, lack of educational materials 4% are major reasons to flee to the street [3]. The economic factors such as lack of enough food 43% boys versus 59% girls. Moreover, the need to contribute their family financially (74% boys versus 81% girls) joined the street. The research conducted in Khartoum, Sudan, shows that the pulling factors like peer-pressure (84%), city life glamour 76% bored at home, 79% forced, and peer influence peer influence 8.3%, them to join the street society [4].

Worldwide, street children have been under-represented for too long in health research, care, support and protection. This amplifies the plights among this minority group. The systematic review of Low and Middle-Income Countries (LMICs) illustrates street children survival behaviors resulted in disproportionate morbidity i.e. infectious diseases, harassments, child trafficking, sexual assault, and to a lesser extent growth problem [5]. The study conducted in India shows majority (92.5%) of street children have experienced physical harassment [6]. About 90% of street children experienced sexual violence, sexual exploitation (50%), unwilling touch of child’s genital area (80%) and physical hurt (80%) [7]. The research evidence conducted in Egypt shows that about 93% of street children have experienced harassment or abuse by policemen or other street children. About 62% have used drugs and 67% of them engaged in sexual activity [8]. Within the age, range of 15–17 years old about 54% are reported multiple sexual partners-where 52% of them never used condoms. Most girls (53% in Greater Cairo and 90% in Alexandria) have experienced sexual abuse in their life span of street [8]. Furthermore, about 40.8% of females and 28.5% of homeless boys have experienced with verbal harassment and about 34.2% of women and 21% of men experienced physical assault within the past 6 months of the study. Another study conducted in Kenya shows that bullying is the most common where it accounts for 87.9% of street children [5, 9].

Ethiopian street children are facing many health and social problems too. The research evidence in Ethiopia show that street children are at high risk of sexual and physical exploitations and they are 2.5 times more likely vulnerable to HIV and STIs [10]. More than 869, 567 (age 10 to 14 years) children illegally migrated abroad (particularly to Saudi), 70% of girls are sexually abused and 30% of girls raped before reaching the age of eighteen. Moreover, about 48.6% of those below 15 years are engaged in child labor [11]. Alongside, lack of shelter magnificent any type of abuse-where, both sexes have the same sleeping quarter and it is being a critical factor for unwanted early sexual practice. Moreover, the plights of street life push them to turn into sex work and mark rampant sexual harassment among street dwellers and outsiders [12]. In Ethiopia, evidence shows that 75% and 85.7% of street children have had multiple sexual partners and practiced survival sex, respectively [13]. The study shows that about (46.4%) of street children have practiced sexual intercourse before reaching the age of sixteen, of which, 32.2% forcefully raped, 17.4% started to drive social connection [13]. Despite such practices, condom use practice (14.3%) among street children is very poor [10].

### Social protection services

The social protection (Social security) is one of an important component to poverty reduction strategy and reduces vulnerability to economy, social and wellbeing among street children [12]. However, the research conducted in Uganda shows that very few social protections exist, this service hardly accessed, and in some cases, the service have accessed through the third parties. Moreover, there are no preventive measure services except for rescue, rehabilitation and reintegration [13]. No specific social protection programme targeting street children, considering their unique vulnerability as a preventive measure [13]. The World Bank institute report shows that goodwill alone cannot guarantee a positive impact that lasting solution on the lives of street children. Only focusing on the assistance is as an effective and even can worse the problems, by increasing the child’s dependence on the charity and destroying its incentive to leave the street [14]. Hence, the street children problems could be addressed through health and protection policies and preventive programs that strike at its social and economic causes of flee to the street [14].

### Health policies, programs and political will

Health policy making and political will are another theme that reduce and prevent the street children plights [15]. To have concrete and inclusive health policies that address preventive, reintegration, protection, corrective and reconstructive approaches [16], arriving on the watertight definition of street children is imperative. Despite the fact that there has been a major inconsistency in defining the term “street children”, where many organizations define in different ways- which negatively hummer care and support of this needy minority group [17]. There is variation in defining the term street children since they are not a homogenous group and the way they use the streets widely varies, and challenging to arrive at watertight definitions [18, 19]. The legal social defense department and various legislators consider them as children exposed to delinquency [17]. While, researchers and Non-Governmental Organizations (NGOs) define- children less than 18 years, who spend all or most of their time on the street, who maintain minimal contact with their families, or have no contact at all, with critical lack of supervision, protection or guidance, which makes them vulnerable to a wide range of health and psychological, hazards [20]. However, the United Nations Children’s Fund has labeled them as children in difficult circumstances, which represent a minority population that has been under-represented for too long in health research [18]. However, in this study, street children are definid as the children less than 18 years, who spend all or most of their time on the street and facing the widespread challenges like healthcare disparities, fragmented social network, child trafficking, harassment, and critical shortage of coverage of basic needs.

Despite these difficulties, studies indicate that the government has ignored the problems of street children and community too. This is causing a devastating impact on the development of African nations. Governors’ poor political value results in-where, families are being a major cause of problems and schools are becoming centers of violence and crime and increasing the numbers of street children [21]. Furthermore, poor program design, helpless implementation of policies, strategies, and less political value for preventive interventions are exacerbating the problems. Therefore, poor program design and less political value result in the helpless effort, and failure holistically to address and prevent plights of street children [18, 19]. By most governments in Africa and elsewhere, the traditional response to street children’s plights has been repression and they failed to offer any viable alternative. Hence, politicians, policymakers, and urban planners seem to be helpless in their efforts to either resolve the problems or assist street children and being futile conduct to prescribe realistic and concrete solutions, to date [21]. The Ethiopian government is not exceptional- being; street children are suffering too from similar challenges. Available programs were poorly designed, with less political value worsening the problems [18]. Therefore, this study sought to explore the deeper insights of street children experiences in relation to factors that lead children into the street, street life challenges, means of survival, and perceived strategies to alleviate the plights among street children in Ethiopia. The study aimed to examine factors that lead children into the street, street life challenges, means of survival, and perceived strategies to alleviate the plights among street children in Ethiopia.

**Fig 1:**
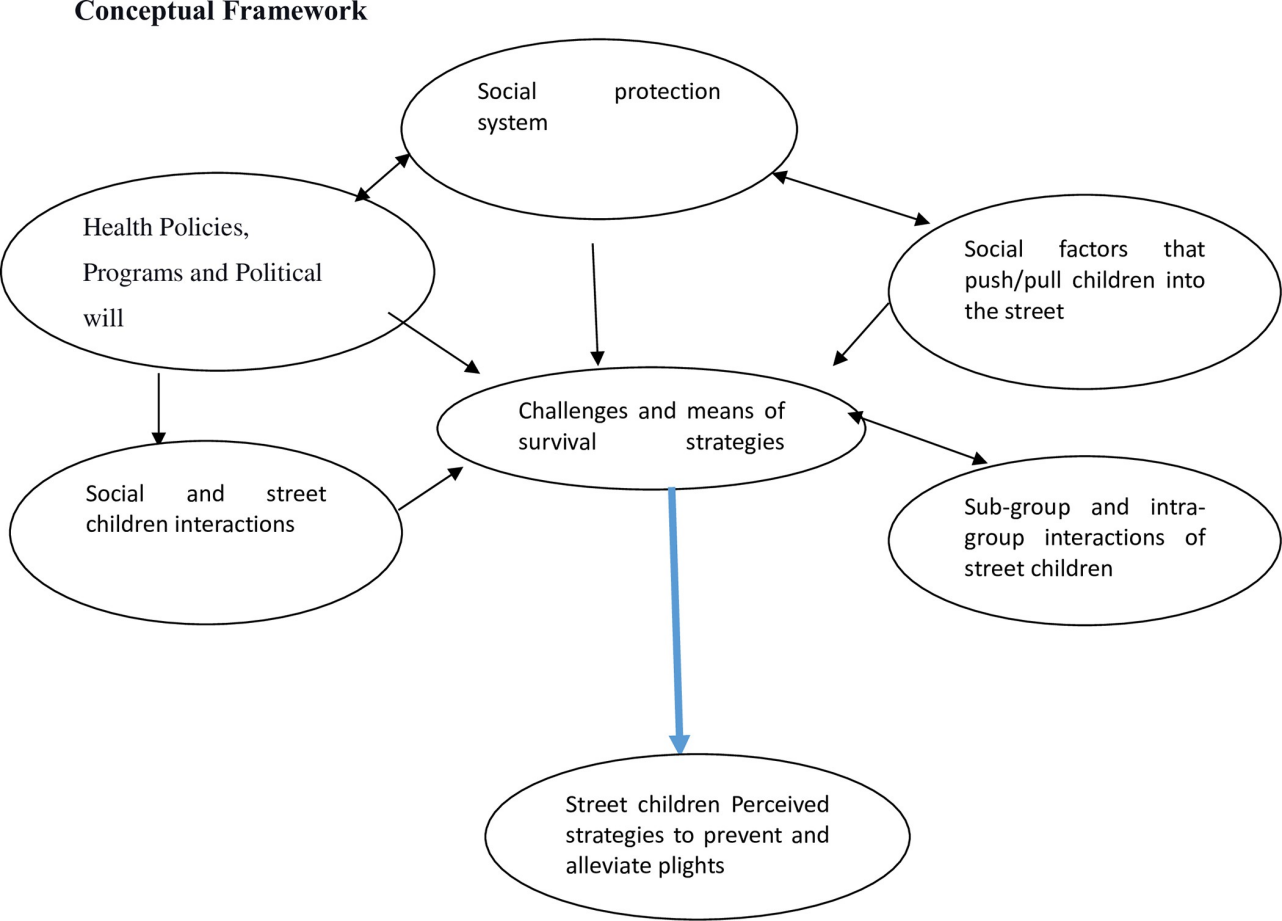
Conceptual framework adopted from Tajfel’s social theory and its application to understanding Metis as a social identity (Jennifer Dawn).

Research departure questions what are the factors that lead the children into the street? What are the major challenges of life in the street? How street children cope up the challenges? What are street children perceived strategies to alleviate and prevent the plights of street life (Fig 1).

## Materials and methods

**Fig 2:**
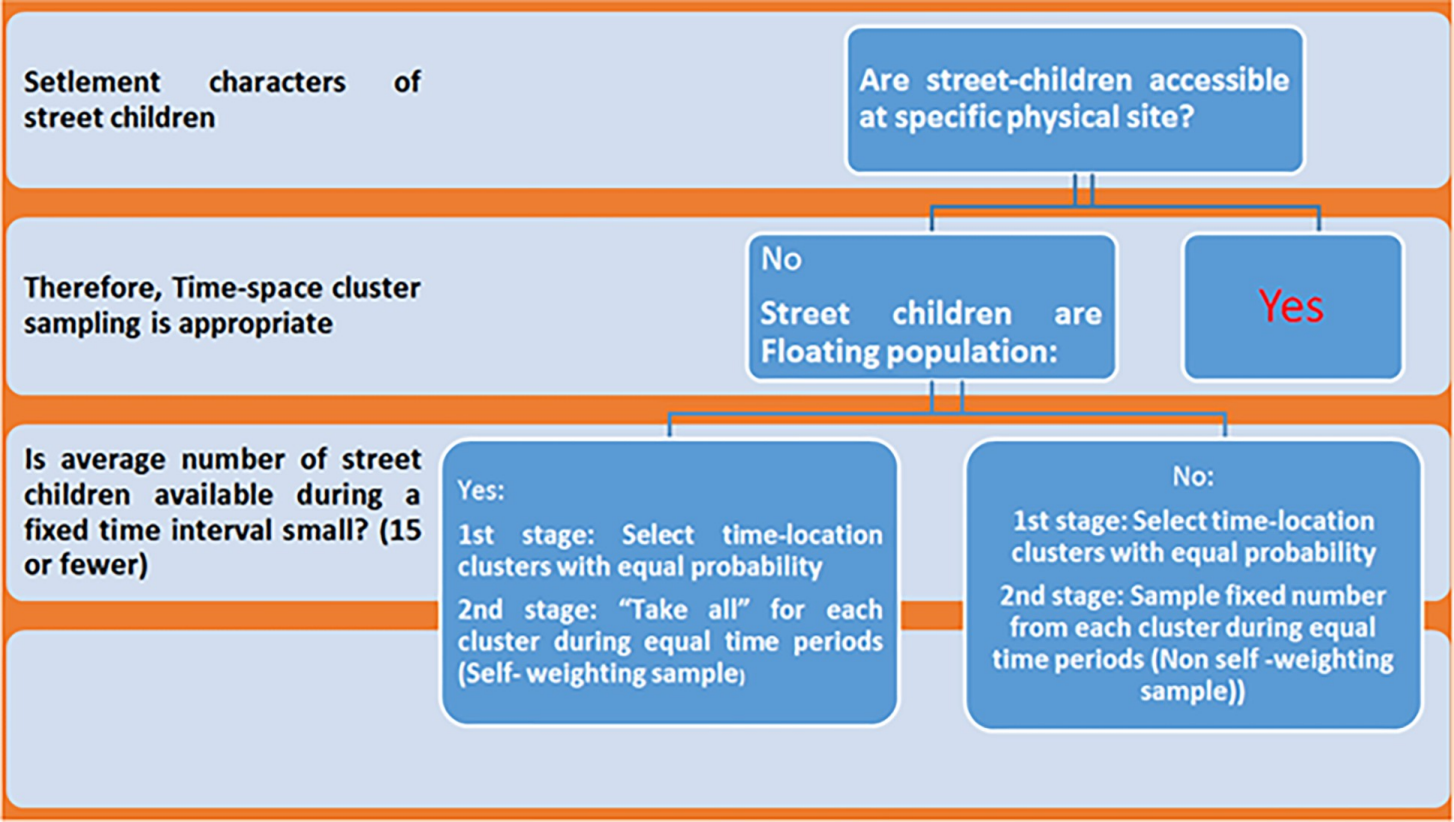
Sampling technique of floating population adapted from repeated behavioral surveys in populations at risk of HIV, Family Health International, 2000.

A Phenomenological qualitative approach was underpinned, since this study approach is excellent method to examine lived experiences of street children. Therefore, the study aimed to explore the deeper insights of street children’s experiences concerning initiation into the street, challenges, means of survival, and perceived strategies to alleviate plights of street life. A Time-Space-Sampling (TSS) was used to recruit participants into the study since study subjects are hard-to-reach populations. First, locations (7 sub-cities) were selected (3 sub-cities for FGDs and 4 sub-cities for interviews, separately) randomly from the sampling frame and participants’ are recruited by self-weighing and non-self-weighting sample during the fixed time from each cluster (Fig 2, sampling frame). All street children age ranged from 10 to 18 years were eligible to participate in the study. The initial plan was to conduct 15 in-depth interviews and 5 FGDs. However, recruitment continued up to data saturation was reached. Where, information saturation was determined by the redundancy of responses, adding more participants into the study did not result in obtaining additional perspectives of the required information, sub-group homogeneity, and data collectors’ expertise considered. Thus, a total of 26 individual in-depth interviews and 8 FGDs (5 FGDs among males and 3 FGDs among Females, separately) were conducted (Table 1).

**Table 1:** Demographic characteristic of street children in Addis Ababa, Ethiopia 2019.

Used research tools FGDs	No of participants	Sex		Educational background			Years lived in the street				Schooling Status		Marital status	
		Male	Female	Never had been to school	Primary (1 to 6)	Junior 2ry education (7 & 8)	Newly joined	One year	2–5 yrs	5+ yrs	In	drop	Single	Informally married
Male FGDs														
FGD1	9	9	-	4	3	2	5	2	1	1	-	9	5	4
FGD2	11	11	-	6	4	1	4	5	2	-	1	10	7	4
FGD3	8	8	-	3	5	-	2	3	3	-	-	8	6	2
FGD4	12	12	-	5	6	1	3	6	1	2	1	11	7	5
FGD5	10	10	-	4	5	1	3	4	2	1	-	10	6	4
Female FGDs														
FGD6	8	-	8	5	-	4	3	2	2	1	-	8	5	3
FGD7	10	-	10	6	-	2	4	4	1	1	-	10	6	4
FGD8	9	-	9	6	-	3	3	2	1	3	1	8	6	3
Total	77	50	27	39	5	24	27	28	13	9	-	74	48	29
In-depth interviews														
	26	15	11	15	9	2	6	5	8	7	2	24	15	11
Total	103	65	38	54	14	26	33	33	21	16	5	98	63	40

FGD n = 77 (n = 50 males and n = 27 females), In-depth interviews n = 26 (n = 15 males and n = 11 females).

The study used a pre-tested interview guide to ensure that questions were unambiguous or not to gather intended information. A pre-test was conducted on 10% of the initial plan, and based on the results some questions were adjusted into the easy way for understanding. Two supervisors and six certified assessors of middle health professionals (bachelor of nursing and health officer) were recruited for data collection, where all of them attended a two-day training program in Addis Ababa, Ethiopia. The training focused on the purpose of the study, ethical principles, sampling, interview techniques, how to take field notes, and audio recording. Data collection was conducted from January to March 2019. Before conducting the interview, data collectors explained the purpose of the study, and all participants assured that their information be kept confidential. Data collectors obtained written assent and consent from study participants and guardians (city mayor and/ or non-governmental organizations i.e. hope enterprise). All in-depth interviews and FGDs were conducted in Amharic in a private place (city hall, church and youth centers) for the purpose of concentration and privacy. FGD participants are categorized into two based on their biological sex; to let them disclose their physical and sexual experiences in detail and freely. Study subjects who have participated in FGD were excluded from in-depth interviews to allow let interviewees come up with additional information and respond to the required information to get additional perspective into the study. Data collection lasted for an average of 30 minute for in-depth interview and 40–60 minutes for FGDs. Interviews were conducted by using a tape recorder up to the level of information saturation. Data quality maintained by field supervisors through wrapped up of recorded data to ensure its completeness and clarity.

The field note memos and audiotaped records were transcribed into Amharic language and translated into English language by using a verbatim transcription. The translated data re-checked by the researcher by listening to audiotape records. Transcribed interviews imported into framework analysis software for analysis. Data analyses conducted in three stages: In the first stage, an analysis of framework was developed and the codebook organized under three broad themes (initiation into street life, means of survival, and perceived strategies to alleviate its challenges), subthemes and categories were added after reviewing participants’ responses (Table 2). In the second stage, the researchers conducted coding by an iteration process, and many more elements related to study objectives were added to framework analysis. In the third stage, the final themes were named, and defined. Finally, results were written by summarizing, interpreting, and quoting, when needed.

**Table 2:** Major thematic areas, sub- themes and categories of in-depth interviews and FGDs responses of street children in Ethiopia, 2019.

predominant thematic areas	Sub-themes and Categories
Theme 1: Initiation into the street and challenges of living in the street	1.1. Pushing/pulling factors into the street life 1.2 Challenges of living in the street I. Lack of social ties and networks II. Critical shortage of coverage of basic needs III. Physical, sexual and verbal harassments IV. Child trafficking
Theme 2: Means of survival and coping mechanisms	2.1. Formation of group life versus collective security I..Inter or intra group conflict of interest 2.2. Sharing available resources and vital information 2.3. Sex as a means of survival
Theme 3: Government and NGO structures to available resources and perceived strategies	3.1. Available government and NGOs programs and initiatives to halt the plights of street children 3.2. Street children proposed perceived strategies to alleviate and prevent the occurrence of plights. I. Separate shelter to spend over the night II. Regular health education, health services and life coaching III. Effective implementation of child protection policy IV. Creation of income generating activities for self-help V. Community strengthening as a preventive strategy VI. Reintegration with families or extended relatives

Ethical clearance was received from the Ethiopian Public Health Institute (EPHI) review board. The EPHI review board also approved obtaining consent from guardians or non-governmental organizations (NGOs) on behalf of the minors that did not have legal parents or guardians. Further permissions were obtained from guardians or non-governmental organizations (NGOs) that are working with street children and had jurisdiction to provide informed consent on the behalf of the minors in the study. Since the study participants were younger, written assent was taken from the study subjects. To compensate for participants’ time, the researchers provided 50 ETB (1.9 USD) per person. Participation in the study was voluntary and information collected from the study subjects was handled confidentiality.

## Results

### Demographic characteristics of study participants

As shown in Table 1, about 103 (n = 65 males (M) and n = 38 females (F)) of street children (age 10–18 years) took part in the study, where 26 (n = 15 M and n = 11 F) are interviewed and 77 (n = 50 M and n = 27 F) participated in (FGDs). Of the 103 participants, 39 never have been into schooling and 59 (57%) of participants dropped their education; whereas, 5 (5%) were into schooling by in and off style. About 40 (39%) of participants are “married” informally, to one another to cope up with the challenges of street life. Thirty-three participants newly joined the street society, while 16 participants lived for more than 5 years (Table 1).

## Theme 1: Initiation into the street and challenges of living in the street

Initiation into the street is a rite of passage marking entrance and acceptance by the societies of a street and practicing its culture and work to survive (Table 2). It might be joining either alone or as a group into the street society due to a variety of push/pull factors. When one joins, he/she would be introduced to the hierarchy of sub-group structure, line of command, power, sex, loyalty, role, and responsibilities of the group members, since it is the beginning of new life. Five of the 8, (62.5%) of male discussants reported group joining has a great advantage over joining as a single person into the street society. When you join a group, you do not have the problems of socialization with other street dwellers. You might not face the harassment of the first-day arrival by other groups or individuals.

*“…*.*Especially*, *if you have one or a more experienced street child in the group*, *you will get the information how to live in the street*, *how to search for basic needs and security issues in the street*. *Such grouping is great that can survive and protect group members from any type of harassments*” (male FGDs).

Participants reported that, if you joined the street society as a single person, your fate is on the hand of strangers that you met in the street. They have power over your body and can do what they like. On arrival, you don’t know with whom you are, with whom you have a sleeping quarter. Most of the time, group sexual intercourse is common practice for newcomers. If you reject their idea, you may be beaten-up, and chased-away from that particular area. Thus, the first-day initiation into the street is the most difficult as to their experiences (male FGDs). A fourteen-year interviewee said that he arrived at Addis Ababa around the bus station at 6:00 PM. While he was looking for shelter and something to eat, the time came to 8:00 PM. He said, one of the street boys told him to have sleeping quarters together. When he heard this, he was very happy. He gave him a loaf of bread, and took him to the railway building, where, he has been sleeping. He shared night cloth and sleeping quarters with him and have a sleeping quarters together.

“…*Just after an hour*, *he forced me to have anal sex*. *I swear to St*. *merry*, *he had anal sex with me and continued it over the night*. *Starting from that day*, *I lost confidence in my sexual identity*” (male interviewee 7).

A female interviewee pointed out that her mother was late and she is the only child in the family. Since the death of her mother, her father drank alcohol every day. Later on, he forced her to have sex with him. Always, at night, he drank alcohol, back to home, and forcing his daughter to have sex. “*Hmmm…forcefully*, *he had sex with me many times*. *This situation left me with psychological pain*. *One day*, *I decided to escape from home to join the street society*. *Then*, *I fled into the street”*.

Then, on her first day of arrival in Addis Ababa, she met one older street boy. He greeted and talked to her. He said, I think you seem a new person to this area, she said yes. He requested her to stay with him, and she said, where? He told to her, as he is street boy and living under-railway building.

*“…*..*I said alright*. *However*, *now I am very hungry*, *would you give me something to eat*? *He runs away*, *and brought me bread*. *Then*, *I decided to be with him*. *We shared his night cloth and sleeping quarters*, *where we enjoyed sex over the night*. *With him*, *I have been two years in the street as my informal husband*. *Sometimes*, *I am practicing sex work and have paid sex*, *to survive*. *Know*, *I felt the ideal freedom*. *Even though*, *I am selling my flesh to survive and facing many derogatory and abusive words in the street*. *Despite this*, *I am psychologically free*, *compared to the previous time that I faced at home*. *Imagine*, *having sex with your father*. *Hmm…*.*in my life*, *I don’t forget its psychological pain forever”* (Female interviewee 17). Similarly, two female interviewees reported the same tragedies (interviewees 19, 26).

The 4 of the 8, (50%), male discussants pointed both male and female have the same sleeping quarters-which, promising for group sex. New arrivals used as sex object, by older boys and shopkeepers’/ institution guardians.

“…*The psychological trauma of post-anal penetration is the most painful that you cannot forget*. *You may think about it repeatedly*. *Even*, *you may face mental health problems like hopelessness*, *depressive symptoms*, *self-harm*, *and to the worst suicidal attempt*. *To be out of it*, *you may start drugs and get out of its pain*. *Gradually*, *adopt the pain of anal penetration and form harmonized relation with friends in the street*. *The situation is re-cycling*. *When you become senior*, *you may do the same thing to new comers and can’t be out of this circuit”* (male FGDs).

### Sub-theme 1.1: Pushing/pulling factors into the street life

Participants revealed the complexity of push and pull factors pose a risk of flee into the street. Pushing factors are any kind of influencing factors that force children to leave/escape from home and flee into the street.

*“…The pushing factors such as poverty*, *separation of parents*, *family death*, *economic decline*, *single-parenthood*, *child abuse*, *neglect*, *school dropout*, *land grab and family eviction due to urban development and family size were the top causing factors of flee into the street”* (male FGD). Following land grab, millions of local communities around capital city (Addis Ababa) have been forced to resettle without any type composition. In such essence, forced eviction of families fueled the number of street children in the country. Moreover, five female interviewees revealed that death of the family, divorce, destruction of extended relatives’ relationship and schooling problem are the most common reasons of flee from home (interviewees). Pulling factors are those that draw/enticement children to flee into the street. The 3 of the 8, (37.5%) of Female discussants reported that “…*Enticements of apparent freedom*, *financial independence*, *peer influence and traditional values i*.*e*. *adventure and city glamour were factors that draw children into the street” (FGDs)*.

### Sub-theme 1.2: Challenges of living in the street

Street children are facing many challenges and difficulties of street life. Discussants reported that when you face any of the pushing and/or pulling factors discussed above, you could not expect good situation to live with your family. The only chance is escaping into the street and enjoys the ideal of freedom. Interviewees noted, living in the street has many struggles, coercions, and maltreatments.

“…*You might be facing many challenges in your street life-span*. *Hence*, *living with the sorest condition is the only chance we have”* (male interviewees). *Reportedly*, *absence of clear and contextualized strategies and policies as-well-as little attention of government to address our difficulties made us highly vulnerable to any kind of problems and exacerbating the plights in the street”* (female FGDs).

#### Category I: Lack of social ties and networks

The five of the 8, (62.5%) of male discussants reported starting from the day of flee into the street, they lost social ties and networks with their families and communities, where they live in.

“…*We are considering ourselves as thrown-away children*. *We are living in ignored world by both our families and communities*. *We have not any social ties and networks*. *We are the most deprived people*, *with no access to family love*, *care and affection*. *Nobody looks after you*. *You don’t have anyone to share your idea with*. *For whom do you cry*?*”* (Male FGDs).

Fourteen of the 26, (54%) participants pointed street children are found instable-where they left family for abuse, community hate them for their dirty lifestyle and consider them as a crime person. Due to this, lack of proper social ties and networks with their family and community is common.

“…*We are alone (no father or mother) who care for you*. *If you try to have healthy social networks with the community*, *the entire of the community hate you*, *due to our dirty living style*, *consider you as criminal and pin-pocket person”*, *(female* interviewees).

Similarly, the 3 of the 8, (37.5%) reported their social tie is hazy and distorted.

*“…Our social ties and networks are distorted*. *You feel hopelessness*, *your current and future life is shadowy*. *We cry out looking for help*. *However from where we get it*? *There is no anyone in our side*. *At times*, *we feel lonely; we suffer from lack of sleep*, *anxiety*, *isolation and mood depression*. *To hide yourself from such conditions*, *we use local alcohol*, *glue*, *hashish and Khat”* (male FGDs).

#### Category II: Critical shortage of coverage of basic needs

Three of the 8, (37.5%) of male discussants reported street children are young people who are living in ignored tragedy with the critical shortage of coverage of basic needs. like other human being, they need basic needs from their biological families to have healthy life, but in their case, they are responsible to look for it.

“…*At this age*, *we are conciliating our schooling and forced to engage ourselves in begging*, *collecting materials from the garbage*, *selling small things*, *manual work*, *prostitution*, *etc*. *You are the only one who act as a father or mother and responsible for daily coverage of basic needs*. *While looking for coverage of daily basic needs*, *we face many struggles and coercion from the street society and/or community”* (FGD). The 13 of the 26, (50%) of participants agreed the most challenge of living in the street is lack of coverage of basic needs. “…*The experience of street life appeared harder than what the community think of it female”* (interviewees).

#### Category III: Physical, sexual and verbal harassments

Five of the 8, (71.5%) of discussants reported street children are living in the world, where they are forced for early sexual initiation, multiple sexual partners, group sex, either of homo or heterosexual. Most of them initiated early sexual intercourse before flee into the street where, there are abusive families or extended families force them and be a main reason to be in the street. Others raped and forced to have sex either anal or vaginal in the street. This situation exposes them for health problems i.e. HIV/STIs, hepatitis, early pregnancy and its psychological trauma.

*“…While living in the street*, *both male and female faces similar physical and sexual violence*. *Either of us have the fate of sexual harassment-which is the worst in the first day initiation into the street society-where you face the most intricacy of either anal or vaginal penetration*. *Anybody*, *either the street boy or shopkeepers/guardians do what they like*. *Both male and female will face this ugly life for advanced protection*. *You will stay in the same sleeping quarter (under railway building*, *veranda etc*…*)*, *where you forced for unwilling practice*. *Such disinclined practices are rampant among us or outsiders”* (male FGDs).

Unprotected sex with strangers is common, where condom use is vested under the interest of strangers you met in the street. Most of male participants have reported post-anal penetration; psychological trauma is always memorable pain, which, you might be victim of it by the first day of joining with street society. When you become experienced, you can do the same thing to the new-comers, if you don’t afraid of your God” (male FGDs).

Nineteen of the 26, (73%) reported “You cannot out of harassments-where, any strangers do it, while you live in the street. Anybody can beat/slap you if you resist any unwilling practice. We are living in ignored world, where there is no any social protection grantee you-which, exacerbate any type of harassments i.e. beating, slapping, sexual violence and even murdering” (male and female interviewees).

#### Category IV: Perception of child trafficking

Even though the root causes for child trafficking are too difficult to know, millions of street children have been experiencing it, at least once in their lifetime. They are vulnerable to trafficking, due to the helpless implementation of social protection policy by the government. Five of the 8, (62.5%), discussants reported child trafficking for cheap child labor and/or sexual intercourse.

“…*We are highly vulnerable to trafficking*. *The trafficking encompasses within the city (Addis Ababa) or out of the city*. *People need you*, *for the cheap uncompensated labor force and sexual activities—where you are forced for cheap labor and/or sexual materialize”* (male FGDs). Two of the 26 interviews reported street children to have limited education and poor socio-economic factors that made them vulnerable to trafficking. A fifteen-year boy interviewee reported “…*I and my friend came from Harargeh (east part of the country)*. *One day*, *two females came and have taken us to their home*. *After we arrived at their home*, *they immediately offer to shower and clothes to change*. *Then*, *they forced us to have sex*. *I have been there for three days and runaway from*. *But*, *my friend is still there”* (male interviewees). Similarly, interviewees (11/26) shared the same tragedies.

## Theme 2: Means of survival and coping mechanisms of street life plights

Street children as much as possible prefer and reside in areas with a special supportive environment and characteristics that do not conflict with their life-style, nor pose intimidations against their existence. They usually teach one another how to earn basic need coverage, where to go for living, and what to do in case they face problems, which is a clear exhibition of their sub-group culture. After fleeing into the street, they were forced to have an active role and participate in informal work sectors and survival sex to sustain their life.

“…*We survive through participating in transactional sex*, *scavenging garbage*, *labor-intensive work to change with bread*, *begging food from restaurant/hotels*, *take food from street food vendors*, *sometimes stealing*, *washing cars*, *selling small things like tissue paper and toothbrush*, *carrying luggage and heavy things”* (male FGDs).

Six of FGDs (75%) of discussants reported their lives were full of struggles, coercions, and maltreatments, exploitation, and violence among themselves and by shopkeepers, institution guardians, striders, and hawkers in the street. Seventeen of the 26 interviewees reported living in the street environment places them at special struggles and coercions. To resist such actions, most street children form small groups to counteract negative influences and practices. They are extremely aware of the problematic and often dangerous environment in which they reside. They have an obsessive concern about troubles and remind their group members to be the lookout for situations that might lead to conflict. Hence, they tend to be strict in their practices, encouraging children to respect and participate in sub-group collective security. Newcomers are liable to have taken orientation and direction for the first time of joining the group. In such ways, they use different coping mechanisms to confront the struggle of street life.

### Sub-theme 2.1: Formation of group life versus collective security

The journey of living in the street have full of struggles and coercions among themselves and outsiders. In the street society, many things are uncertain and you live in the victim of violence. To cope-up with such encounters, they form a small group- where every member of the group has his/her role. The 6 of the 8 FGDs pointed to cope up with such a harsh environment, forced to form a group-which, enable them mutual support and protection with a strong sense of companionship with strong group norm. The street children were governing their interpersonal behaviors, and manage violence by a, informal rule. The rules prescribe both proper comportment and a proper way to respond if challenged. They regulate the use of violence and allow those who are inclined to aggression to precipitate violent encounters in an approved way. The rules have been established and are enforced mainly by a well-experienced street-oriented child. Everybody knows that if the rules violated, there are penalties.

“…*Street world is characterized by miserable deprivation*, *subjected to physical*, *verbal*, *and sexual abuse*, *and victims of violence*. *We are forming a group*, *and trying to resist any violence in the form of group security*. *We have a group norm and culture*, *which led by one of the brilliant street-oriented children*. *Through forming group security*, *we protect ourselves from any coercion*. *Within the group*, *there is sometimes intrapersonal violence*, *most likely by the older street children*” (male FGDs).

#### Category I: Inter or intra-group conflict of interest

The 5 of the 8 male discussants said most of the time, the group might be dysfunctional and disperse its members. Many things are uncertain about how long we are living, and we believe we may die due to violence among ourselves or by violent strangers at any time. They accept this fate and living on the border. Anybody can intimidate you; we try to counteract it. During this time, you do not know what will happen to you.

“…*We discourage violence as a primary solution to resolving disputes and encourage the group members to accept nonviolent behavior*. *Nevertheless*, *if the negotiation goes down*, *every group member runs for self-defense*. *Many of us*, *much more concerned about the threat of our group norms*. *If somebody breaks the norm and culture*, *fighting within the group is common practice*. *Even though the nonviolent orientation rarely overcomes the impulse to strike back in an encounter*, *it may lead to a certain confusion and lead to profound violence among the group*. *Hence*, *member of a group strives to go for bad action against each other” (male FGDs)*.

Moreover, female discussants reported females were mimicking the male behavior and trying to have their version of manhood.

“…When you act as manhood, you get respect and will be recognized by the group members. We try to achieve this in the ways that established by the boys and doing what the male street child do i.e. using abusive language, to be recognized person to actively participate in resolving disputes within the group or between the groups” (FGDs).

Five of the 8, discussants pointed conflict among street female exists due to the assessment of beautiful girl within the group i.e. which girl in a group is the most beautiful and competition over boyfriends within the group or from other groups.

*“…The main cause of conflict within the group is he says*, *she says rumor*. *Usually*, *one girl might be said something negative about somebody in a group*, *behind that person*. *The negative saying will back to the person who talked about him/her*. *In such essence*, *might be led to group gossiping*, *which can be the main reason for the group violence and disperse” (male FGD)*.

### Sub-theme 2.2: Sharing available resources and vital information

There is compassion and caring culture among the sub-group of street children. The sympathy kindness and devotion of sub-group members of street children is astounding and astonishing. All of the discussants noted everybody within the group has his/her mandate to look and search for daily basic needs. Some of the individuals participate in manual work, scavenging through garbage, begging, selling small things- whereas, females might be taking the responsibility to have survival sex and bring the money for the group.

“…*Sharing resources and information is our fundamental activity*. *We have our sub-culture*, *which gave us a group identity*. *The group has its own rule to welcome newcomers to the group with the mandate of orienting survival skills and socializing with the group*. *New arrivals oriented about means of survival and as streets are full of drugs and violence”* (male FGDs).

The 17 of 26, (65%) of interviewees pointed, “…*To survive this harsh world*, *we are engaging ourselves in all kind of activities that bring money*. *At this age*, *we are engaging ourselves in manual works like shining of shoes*, *pushing trucks and gathering garbage and carrying it to the dumpsite*., *In addition*, *selling rubber bags*, *tissue paper*, *and toothbrushes in the street*, *scavenging garbage*, *and engaging in paid sex*. *To survive in the street*, *you engage yourself in any type of activities and ensure the group self-support”* (female interviewees).

Six of the 8, (75%) discussants pointed out that sharing vital information is the most important and imperative activity to survive. They move here and there to settle down in areas-where, they feel secure and gives them the possibility of earning coverage of basic needs and able them to have fun with the group members. They prefer popular, market and commercial areas to be engaged in the informal work sector-where, they earn living and enjoyment expenses. Such areas would be identified, and the group would be informed via information sharing.

*“…You know*, *there are interesting kindheartedness and devotion among group members*. *You share important information to earn living and enjoyment expenses and bring it for the group members*. *You are responsible to bring money from any activities you get and share with your group members”* (male FGDs).

### Sub-theme 2.3: Sex as a means of survival

Many research pieces of evidence show that street children are a shift to sex work as a means of survival. All discussants pointed most of the street children turn to sex work to survive. Both males and females are highly at risk of participating in unwilling sex. Where unprotected sex with strangers is common practice and made them vulnerable to health problems and early-unwanted pregnancies.

*“…To survive*, *we engage ourselves in sex work*. *Sex work is the main income-generating activity for our informal wife*. *Over the night*, *if you see the streets*, *it is congregated with many young women*. *Most of them are our informal wives acting as sex workers women and seeking paid sex*. *When she back to act as a wife*, *we share the money and expense for our basic needs*. *If she has an informal husband within the group*, *her husband is the one who shares the money with her*. *If the group lack coverage of daily basic needs*, *her husband is responsible to buy food for that particular day”* (male FGDs).

Eleven of the 26, (42%) participants reported, if the expense for survival is very tense, boys forced to have paid sex. Sometimes, boys engage themselves both in homo and heterosexual intercourse, due to the immediate need for basic needs.

*“…With outsiders’ paid sex is common*, *if other means of survival are unsuccessful*. *Our intention is on the money rather than protected or unprotected sexual activities*. *But*, *if you have paid homosexual activity*, *its psychological pain was the most puzzling to categorize your sexual identity”* (male interviewees).

## Theme 3: Government and NGO structures to available resources and perceived strategies

The twenty-first century presents a hostile environment to many millions of street children in many African countries that need attention by both government and NGOs to overcome the challenges. Such organizations have a responsibility to eliminate all kinds of human right violation, discrimination, improve access to education, health services, and coaching of this marginalized minor population. Furthermore, community members have a responsibility to support and advocate for the rights of street children and the government should also encourage NGOs to have a child-rights focus.

However, for most governments in Africa and elsewhere, the traditional response to street children plights has been tyranny and failed to offer any viable alternative. Hence, politicians, policymakers, urban planners, and NGOs seem to be helpless in their efforts to either resolve the problems or assist street children, and being futile conduct to prescribe realistic and concrete solutions.

### Sub-theme 3.1: Available government and NGOs programs and initiatives to halt the plights of street children

Despite these facts, the government and NGOs failed to implement the policy due to several factors i.e. designing problems of policies, strategies, and programs, budget allocation, poor inter-sectoral integration and lack of comprehensive standards were bottleneck of policy implementation. Furthermore, fewer and/or no political value for such victimized of young people is exacerbating the problems [22]. Consequently, street children have been considered as a felon, outlaws, and having a mentality of committing a crime by both law-keeping bodies and society. This, in turn, affecting this young person to be accepted by their community and get legal protection under the law-which, which aggravate the problem against street children in Ethiopia.

*“…We are human beings with no access to legal protection*. *We are forgotten groups by the government*. *The police officers always harass*, *beat*, *and jail us without any evidence*, *because they don’t think of us as a normal person with legal rights*. *Among us*, *if someone goes to prison for a small crime or just with suspicion*, *it will be difficult to get him/her to bail out*. *No one cares or trusts us*. *We stay in prison for weeks or months*, *whether we do crime or not*. *We are just people with no legal protection services”* (male FGDs).

Most of the interviewees (19 of the 26, 73%) reported, *“…Government doesn’t know us*. *Sometimes*, *we hear things over media about street children*, *but none of the government body intends to do practical and reliable activities to respond to our problems*. *They just talk about what they felt*, *but they don’t know a bit about our challenges”*.

They do not offer any viable alternative solutions. They failed to set down realistic and concrete solutions for our problems. On the other hand, police officers are our enemy. If something happens, we are their first option and victims of their ruthless beating.

*“…We are just a bunch of criminals in their eyes; who happened to be outlaws and just hate being legal*. *Hence*, *they beat us like dogs*. *How can we think that the government knows us*, *while their people treat us like animals*? *It is ridiculous*! *Even animals have the right*, *but us…*. *Sometimes*, *society is better*. *At least*, *we hustle and make our daily living*, *because they let us*. *Some good people help and provide us with different things”* (female interviewees).

Most 5 of the 8, (62.5%) of discussants reported the absence of clear and contextualized strategies and policies as-well-as little attention of the government to address and prevent our problems, making them more vulnerable to inhumanity practices. Sometimes, their problem was individualized, depending on a personal history that made him/her flee into the street and personal status at home and living in the street. *“…The care of street children must be effective through addressing the personal situation by categorizing sub-group experiences and holistically address the categorized problems*. *To do this*, *studying child profile and categorizing similar profiles and then*, *strategically attempting to solve is imperative for us*. *Therefore*, *a better understanding of children living and growing up in the streets is essential”* (female FGDs).

### Sub-theme 3. 2: Proposed and perceived strategies to alleviate and prevent street life plights

This qualitative inquiry addressed street children’s perceived strategies that support the development of clear, comprehensive, and contextualized strategies to respond to difficulties of street life. This part of the study aimed to look at the street children has proposed strategies to discourse the plights against this young marginalized group. Their perceived strategies will benefit in recommending the Ethiopian policymakers and urban planners for urgent and sustainable interventions.

Participants listed the following strategies to be considered by the government and NGOs, communities, and other interested stakeholders’ to effectively and efficiently address and prevent the plights against them.

#### Category I: Separate shelter to spend over the night

The Ethiopian social protection policy states that every vulnerable child is liable to have shelter, with very little and/or no implementation. Hence, the absence of shelter imposing both males and females to have the same sleeping quarters. They are forced to share veranda, railway cove, and open space, etc. by group altogether. Six of the 8, (75.5%) discussants reported whatever the reason, being the street child is sleeping in insalubrious places, facing violence and becoming an expiatory victim of it. The street environment does not offer any social protection or shelter. Hence, they are victims of any type of harassment, cold and rain, since there is no safe sleeping quarter. The mainstreams of the street children are living in conditions of severe deprivation and unsafe environment-which place them at all kinds of risks.

*“…We are living in a harsh environment and with no shelter*, *where male and female share the same sleeping quarters*. *This situation amplifies sexual and physical violence among outsiders and us*. *If the government can hear us*, *we are loudly shouting out and looking for service of separate and safe night shelter”* (female FGDs).

Twenty-three of the 26, (88.5%) interviewees pointed to lack of night shelter in the street as providing an enabling environment for prostitutes-where, an older street child, night guardians/watchmen forcibly engage them in unprotected sex.

*“…We are begging for multicultural approach practices that might be amenable for the provision of night shelter*. *It benefits potentially to reduce the risk of harassments*, *cold and rain that we are suffering from”* (male interviewees).

#### Category II: Regular health education, health services, and life coaching access

Street children’s access to regular health education, healthcare, and life coaching services are the backbone to empower, address, and prevent plights. Life coaching is a motivational and behavioral change approach that helps people to set and reach better goals, leading to enhanced well-being and personal ability of functioning. Since, they are highly exposed to verbal, physical, and sexual violence and psychosocial difficulties, which might be mitigated through the assistance of caregivers (assigning volunteer ambassador mothers/fathers)—who have an interest and capacity to parent and support this young person by life coaching. Evidence shows that life coaching is a promising behavior change approach for empowerment, efficiency, and well-being of domestic violence survivals.

Nineteen of the 26, (73%) of interviewees reported access to regular health, education, and life coaching services are essential to make street children healthy, educated and allow to a healthy and happy lifestyle. Due to the absence of guidance and their lifestyle, the health of street children compromised from day- to-day.

“…*We …*. *Street children do not have access to sanitary facilities that made us often dirty and infested with fleas*. *Due to the lack of hygiene*, *we exposed to different infectious diseases* (female interviewees).

Five of the 7, (71.5%) of discussants pointed poor living styles have a negative impact, not only on their physical and psychological development but also their socio-economic development and social networks.

“…*We are looking for any organizations or individuals*, *who make our education reality and who can help us to access health services and able to coach our living styles*. *If we are aware of the ways of infection transmissions*, *we recognize them and protect ourselves in the street life span*. *Not only this*, *such services are essential in increasing our socio-economic productivity as-well-as important in determining our future life”* (male FGDs).

#### Category III: Effective implementation of child protection policy

The implementation of Ethiopian social protection policy would be an important, and should get in need of attention for this young marginalized people. The government failed to provide special legal protection and social services for street children exposing them to child labor, trafficking, violence, and moral problems that contribute to social and psychological predicaments. Twenty-two of the 26, (84.6%) interviewees reported, since street children are a marginalized group of young people, they are often victims of discrimination and facing inhuman practices from day today.

“…*We do not have any human right protection services*. *Implementation of such policy is vital for us*. *We don’t know if government considers us as a human-being*, *we need such practices”* (female interviewees). Six of the 7, (85.7%) of discussants highlighted, community members have prejudices and stigmatizing by naming and calling them as “street child”. They pointed even the name **“Godana tedadary”** which means street child/children and such language itself is a derogatory word that affects their morals. Not only this, such language is prejudiced and spoils our social networking with the community.

“…*Nobody stands behind us to look after or protect our rights*. *Anybody come and abuse you*, *whether physically or sexually*. *If it is possible*, *we are pleading for social protection that liable to our rights*. *Generally*, *we are demanding basic human rights protection and loudly shouting for such services*. *We are looking for any organizations that can ensure our right protection and possibly reduce our level of vulnerability” (FGDs)*.

#### Category IV: Creation of income-generating activities (IGAs) for self-help

Financial constraints forcing street children to conduct activities like heavy manual, sex work, and child labor. Hence, the financial burden is compromising their health, psychology, and schooling. The Ethiopian social protection policy document stated that highly vulnerable children are liable to get IGAs training and provided with IGAs. However, street children responded and witnessed, as they never have seen such activities and practices. All FGDs of study participants were highly interested in the IGAs for financial self-help. Discussants reported their working conditions are more hazardous and less likely to be protected by the employment legislation since they are participating in informal sector activities. A twenty-four of the 26, (92%) of interviewees reported

“… *We are looking for local authorities or international organizations support to establish micro-enterprise and able us for self-help*, *where the government ensures the safety of working environment with protected employment legislations*. *Every day*, *we are participating in informal sector activities-where the employer abuses you and even uncompensated payment*. *Sometimes*, *refusing to reimburse for the activities you have done”*. Six of the seven discussants reported; to ensure financial self-help, creating small micro-enterprises need to be gain attention and access to capital finance operations and training on the specified activities. Such generous support may relieve our financial constraints and basic needs problems.

### Sub-theme V: Community strengthening as a preventive strategy

The one pillar of Ethiopian social protection policy states encouraging community-based social support for vulnerable children through buying school material, food, and shelter. The community care coalitions, district-level social protection committees allowed to collect voluntary contributions and allocate for vulnerable child social protection actions. Such informal mechanisms are important social support mechanisms that bedrock for child protection and empowerment. However, such practices are absent or rarely implemented in the community and contributing to the increment of street children in the cities and urban. [23]

Almost all (24 of the 26, 92%) of interviewees preferred preventive strategies within the community in advance. Preventive strategies within the community are the main concern and have importance to minimize the magnitude of street children in the street. They noted preventive strategies could address the main reason to flee from home before being the main reason.

“….*For our most of street children*, *the main reasons of fleeing into the street are material and financial problems*, *abusive family like stepmother or father*, *poverty and inability to schooling*, *land grab*, *and family eviction are the factors lie behind the departure of us into the street” (*female interviewees).

Six of the 7 FGDs said if the government body and community members detect their problems earlier and monitor them through social welfare within the community, they might not be in the street. Reported social protection committee that funded and fully mainstreamed social welfare programs in the community-where, they can early detect and take measurements against problems that lead into the street. “….*You knows*, *if there are such services in the community for vulnerable children*, *it is possible to address the root cause and the complex challenges that made us the street boy or girl*. *Even these can be addressed*, *by the religious and community leaders-where they can do the reality of our schooling materials*, *allow to enough food and advising our abusive families to not do”* (*FGD)*. Similarly, most (23 of the 26, 88%) of interviewees are perceived preventive strategy as the main solution.

A fourteen-year-old female reported *“…My father was late*. *After a year*, *my mother married another person*. *My stepfather made me always farming and denied me schooling*. *With this hard work*, *nobody cares about me*. *That is why I joined the street*. *Thus*, *if the community members responsibly*, *advocate the right of a vulnerable child and mobilize the community for our rights and schooling materials and encourage to speak out for our problems*, *definitely they can prevent suffers I am facing here in the street”* (female interviewee 25).

#### Category VI: Reintegration with families or extended relatives

Most research scholars support and recommend the value of reintegration into their families or extended relatives. However, most of the study participants denied the value of the reintegration strategy. Eighteen of 26, 69% interviewees and (4 of the 8 FGDs) of male discussants are denied the value of reintegration. “…*Returning to abusive families is not a good option*. *You fled into the street from where you have neglected*, *abused*, *denied schooling*, *farming*, *no enough food*, *etc*., *and back to such families is unbearable*. *From such families*, *you do not expect good things”*.

However, out of the total participants only (8 of 26, 31%) (Male n = 5 and female n = 3) interviewees and (3 of the 8) male discussants agreed positively on the value of reintegration. Even though they have agreed on the reintegration, they highlighted that it should be with the legal background and the families should agree to nonviolent behavior and promise to school with the presence of a witness.

“…*Ideally*, *reintegration is a good solution*. *However*, *if your families do the same thing as previous reasons to flee*, *it hasn’t been sensed*. *Due to this*, *during reintegration*, *our families should promise us nonviolence behavior and provision of schooling materials as well enough food”*.

Even though most of the researchers recommend the value of the reintegration strategy, only a few participants have agreed to it with outstanding preconditions-where, they beholding for forcing legal background to nonviolent behaviors of families and promising to school. This shows that the street children’s reason to flee into the street and life challenges in the street varies-which needs identifying and categorizing problems and address through sub-culture and reason of fleeing and life experiences of street children.

## Discussion

This study provided an opportunity to understand experiences of street children initiation into the street, challenges, and means of survival as-well-as perceived strategies to alleviate associated plights among street children in Addis Ababa, Ethiopia. This study shows that street children are facing challenges of social network fragmentations, child trafficking, sexual and physical harassments and critical shortage of coverage of daily basic needs. As a means of survival, the street children are using available opportunities through forming small groups. The finding reveal that the local, national government and NGOs give little attention to address the plight of street children. Furthermore, stigmas by the community, limited resources, lack of case-based standardized comprehensive programs, policies and poor political value for such interventions exacerbating the problem of street children.

The study revealed that complexity of push and pull factors that pose a risk of flee to the street. Of these factors, participants reported poverty, divorce, separation of parents, death of one or both parents, economic decline, single-parent households, child abuse; neglect, alcohol abuse, school dropout, family size are among pushing factors. Enticements of apparent freedom, financial independence, friendships, and traditional values i.e. adventure and city glamour is among the pulling factors that draw children into the street. Similarly, the study conducted in Ethiopia and Sudan shows that prior hardships i.e. death of family, war, destruction of an extended relative relationship, denied to basic needs, sexual and physical harassments are imminent factors influencing children into street life [2, 11]. The report of global child protection services shows that difference and diversity are poorly tolerated and leading to neglect and abandonment of children in many cultures. Existing social norms and cultural attitudes towards children often allow abuses of children’s right to care and protection. Furthermore, conflict, disasters, food insecurity due to climate change, social exclusion, violence, lack of access to education, exploitative and hazardous child work, migration, and urbanization are major predisposing factors to the initiation of street life [19].

The finding reveal that both of first day’s exposure to street and throughout street life forced into challenges. They are facing the challenges of critical lack of basic needs, shelter, and any type of harassment i.e. verbal, physical, or sexual abuse. Sexual relations i.e. group sex, oral, anal, and vaginal sex are common harassment among street children or with the community. Mostly, such sexual relationships are used as a defense for the newcomers, and as a means of survival. In the case of refusal of such sexual advances, the newcomers were beaten-up and chased-away from that particular area. This study finding is consistent with the study conducted in Malawi and Ethiopia [7, 11]. This finding shows that social network fragmentation from biological families and communities and a critical shortage of daily basic needs, sexual harassments and child trafficking are identified as the main challenges of street children. Inline to this finding, the study conducted in Kenya and Ethiopia shows street children are facing similar tragedies [9, 11].

The study finding shows that street children are living in the world where they are facing day-to-day sexual, verbal and physical harassments. Due to this, they are forced for early sexual initiation, multiple sexual partners, group sex i.e. homo or heterosexual that leads to early pregnancy, health problems and psychological trauma. Particularly, new arrivals are prone to sexual abuse and exploitation by older street boys, group of street children or guards to secure their protection in advance. Thus, both male and female street children are highly at risk of sexual violence. Concurrently, the study conducted in Ethiopia shows that about 27.5% of street children practiced sexual intercourse in their lifetime, of which 44.6% had begun sexual intercourse before the age of fifteen and 75% have had multiple sexual partners [24, 25].

This study reveal that child trafficking is a prominent challenge that occurs either within or out of city for uncompensated child labor or sex. Limited education or illiteracy and poor socio-cultural background are major factors for vulnerability of child trafficking. Report from street children indicates that house detention and enforced sex with strangers or free exploitation of labor are usually their fate after trafficking. Concomitantly, the report of international programmes on the elimination of child labor shows that in Kathmandu, ambitious lies and fake promises to either the children or their parents are ways of wining their heart for trafficking. As a result, at least fourteen street children are forced to child trafficking for the purpose of domestic child labor, where their working conditions at the first distention were unsafe both psychologically and physically. The majority of them did not get enough food or decent place to sleep and paid uncompensated payment [26–28].

This study shows that there is no any structured social protection system in Ethiopia. Similarly, the evidence in Uganda shows that very few social protections exist, and this service hardly accessed and in some cases, the service have accessed through the third parties. Moreover, there are no preventive measure services except for rescue, rehabilitation and reintegration [13]. A finding show that hardly available programs are poorly designed helpless implementation of policies, with less political value that worsening the problems of street children [18]. Concurrently, most governments in Africa and elsewhere, the traditional response to street children’s plights have been repression and they failed to offer any viable alternative. Hence, politicians, policymakers, and urban planners seem to be helpless in their efforts to either resolve the problems or assist street children and being futile conduct to prescribe realistic and concrete solutions, to date [21, 25].

This study shows that street children perceived positively the value of separate shelter, creating income generating activities, regular health education and life coaching, controlling body for safety and right and prevention strategies within the community are perceived strategies to solve the street children challenges. Similarly, Ethiopian policy stated vulnerable children has the right to social services including basic human rights, health care, education and good nutrition, community-based social support, provision with IGAs training and involving them in IGAs and other studies recommend similar strategies to alleviate the challenges of street children [14, 26, 29]. Whereas, this finding revealed that some of study participants were denied the value of reintegration strategy. In contrary of this, dozens of researchers are recommended the reintegration strategy as a possible solution [10, 24, 26, 27].

## Conclusion

The study has explored experiences of street children concerning pushing and pulling factors being a reason for initiation into the street, challenges, and means of survivals in the street. Moreover, the study explored street children’s perceived strategies to alleviate the street life plights. Participants revealed the complexity of push and pull factors pretense the risk of flee into the street. They are facing challenges of social networking fragmentations, child trafficking, sexual and physical harassments and critical shortage of coverage of daily basic needs. As a means of survival, street children are using available opportunities through forming small groups- where, every individual within each group has a mandate for collective security, sharing available resources and vital information that might be useful. Factors i.e. previous aggressive behavior, being victim of physical or sexual, competition of getting the cute girl and drug use behaviors are the main cause of conflict within or among groups.

Participants reported the local, national government and non-government organizations gave little attention to address the plight of street children. Little attention and lack of strategies targeting street children has made them prone to health problems. Furthermore, stigmas by the community, limited resources, lack of standardized comprehensive programs, policies and poor political value for such interventions exacerbating street children problems. Recommendation- call for different stakeholders in supporting to design and implement clear, case-based and contextualized strategic policies to address and prevent plights. Risk reduction programmes targeting street children by multi-cultural perspectives and involving government, nongovernment organizations, community members and parent engagements should have to take into consideration. Identifying street children and its types with respect to life style in order to satisfy their specific needs.

### Limitation of the study

This study employed only street children-where organizations or guardians who are working with this marginalized people were not considered to take part in the study as key informants.

## Supporting information

S1 File (DOCX)

S2 File (DOCX)

S3 File (DOC)

## Abbreviations

ETB: Ethiopian Birr
EPHI: Ethiopian Public Health Institute
FGD: Focus Group Discussion
HIV: Human Immunodeficiency Virus
IGA: Income Generating Activities
LMICs: Low and Middle Income Countries
NGO: Non-Governmental Organization
OVC: Orphan and Vulnerable Children
TSS: Time-Space Sampling

## References

[1] Action for the Rights of Children [ARC], 2001)

[2] Sofiya Endris*, Galata Sitota 2019 Causes and Consequences of Streetism among Street Children in Harar City, Ethiopia Volume: 7 Issue:2 10.7575/aiac.ijels.v.7n.2p.94

[3] Lalor Kevin 2017 Street Children: a Comparative Perspective, Child abuse and neglect, Vol 23 (8), 1999, pp. 759–770. doi: 10.1016/S0145-2134(99)00047-2 10477236

[4] Mary L. Plummeret.al 2007 Beginning street life: Factors contributing to children working and living on the streets of Khartoum, Sudan Children and Youth Services Review 29 (2007) 1520–1536: doi: 10.1016/j.childyouth.2007.06.008

[5] Laura K., Murray Namrita S. Singh: (2012|) A Qualitative Study of Georgian Youth Who Are on the Street or Institutionalized Int J Pediatr. Article ID 921604. 10.1155/2012/921604 PMC3512289 23227056

[6] Jessica w. et.al 2013 the health status of street children and youth in low-and middle-income countries; systematic review of the literature, journal of adolescent health and medicine 10.1016/j.jadohealth 23706729

[7] P Mandalazi C Banda, Umar E (2013) Street children’s vulnerability to HIV and sexually transmitted infections in Malawian cities Malawi Medical Journal (25)-1 PMC3653190 23717747

[8] Demelash H. and Addisie A. (2013), Assessment of Sexual and Reproductive Health Status of Street Children in Addis Ababa, Hindawi Publishing Corporation Journal of Sexually Transmitted Diseases Volume 2013, Article ID 524076, 20 pages, 10.1155/2013/524076 PMC4437437 26316958

[9] Jessica Woan M. D.a, Jessica Lin, et.al, The Health Status of Street Children and Youth in Low- and Middle- Income Countries: A Systematic Review of the Literature Journal of Adolescent Health 53 (2013) 314e321. 10.1016/j.jadohealth.2013.03.013 23706729

[10] Abdul Hai et, (2014) all Problems Faced by The Street Children: A Study on Some Selected Places in Dhaka City, Bangladesh International journal of scientific & technology research volume 3, issue 10,.

[11] Chimdessa and Cheire BMC Pediatrics (2018): Sexual and physical abuse and its determinants among street children: Qualitative study in Ethiopia BMC Pediatrics 18:304 30231892 10.1186/s12887-018-1267-8 PMC6146752

[12] Dercon S (2011). Social Protection, Efficiency and Growth, ‖CSAE Working Paper Series 2011–17, Centre for the Study of African Economies, University of Oxford.

[13] Kakuru Robert, Archangel et.al 2019 Social protection mechanisms for children living on the streets: Perspectives from Uganda Vol. 11(1), pp. 1–11, doi: 10.5897/JASD2018.0523 ISSN 2141-2189

[14] Elena Volpi 2002 Street Children:Promising Practices and Approaches, 37 pages. Stock No. 37196

[15] Fatemeh Abdi et. al 2017 Health policy making for street children: challenges and strategies International Journal of Adolescent Medicine and Health. 2017; doi: 10.1515/ijamh-2016-0134 28817378

[16] Friberg Amanda & Martinsson Viktoria 2017 Problems and Solutions when Dealing with Street Children

[17] Ali Mehdi, Rapid situation assessment of street children in Cairo and Alexandria report, 2010

[18] Dr. Patricia Ray, Corinne Davey and Paul Nolan Analysis of policy and programmes related to street involved children

[19] Cumber Samuel N. and Joyce M. et, al. (2015) The health profile of street children in Africa: a literature review, Journal of Public Health in Africa; 6:566. doi: 10.4081/jphia.2015.566 28299148 PMC5349275

[20] The definition as such correlates with how street children have been defined in previous research, especially Abu El-Nasr, M., 1992; Sedik, A., 1995; Koraim, A., 1998; Hussein, N., 1998, as well as those adopted by UNICEF and WHO).

[21] Paper to be presented to an International Conference on Street Children and Street Children’s Health in East Africa, to be held in Dares-Salaam, Tanzania, April 19th - 21st April 2000).

[22] Nahom Eyasu, 2018 Policy Briefing on Child Protection Policy of Ethiopia in Emergencies Vol. 18 Issue 3 Version 1, Global Journal of HUMAN-SOCIAL SCIENCE: Online ISSN: 2249-460x & Print ISSN: 0975-587X, Sociology & Culture]

[23] Ljungqvist B. Millennium dream campaign, UNICEF Representative in Ethiopia 2007.

[24] Fite A. C and Cherie A. (2016) Risky Sexual Behavior and Its Determinants among Orphan and Vulnerable Children in Ethiopia, World Journal of AIDS, 6, 111–122

[25] Repeated Behavioral Surveys in Populations at Risk of HIV, Family Health International, 2000

[26] jarosz J., 2016 what is life coaching? an integrative review of the evidence-based literature inter. Journal of evidence based coaching and mentoring, 14(1), 34–56

[27] Vashti B., kath w., et. al 2019 journal of family violence assessing the feasibility of a parent life coaching intervention to support parents and children who have experienced domestic violence and abuse

[28] International Programme on the Elimination of Child Labour, (2002) Trafficking and Sexual Abuse among Street Children in Kathmandu, no 1; IPEC: Trafficking in Children-South Asia (TICSA), First Published

[29] Ethiopia national social protection policy report, 2012.

