# Supplementary material for: Initiation into the street, challenges, means of survival and perceived strategies to prevent plights among street children in Addis Ababa, Ethiopia 2019: A phenomenological study design
Source: PLoS One. 2022 Aug 29;17(8):e0272411. doi: 10.1371/journal.pone.0272411 (PMC9423604; doi:10.1371/journal.pone.0272411)
Supplement: S1 File — (DOCX) [file pone.0272411.s001.docx]

***Brief information for focus group and in-depth interview study participants.***

Hello!

My name is _______. I am data collectors for the research titled with Initiation into the street, challenges, means of survival and perceived strategies to prevent plights of Street chillaren, in Ethiopia. This research is aimed to explore the experiences of street children regarding to the reasons that force you to flee to the street, how you initiate to join street society, the challenges and means of survival in the street and your perceptions how to halt and prevent the street life plights. The finding will help the government, communities and NGOs to develop health policy, child protection strategies to halt and prevent the street children plights. Moreover, stakeholders can use to develop and implement policies and strategies in improving the health and wellbeing of minor underprivileged group of children. You invited to take part in the study. Your kind experiences, opinion and proposed solution will help us to conduct and generate this research finding.

We collected the signed consent form from municipality office who are responsible to look after you. know, if you agree to take part in the study, I am kindly requesting you to sign this assent form and one copy for you and one copy for the PI researcher. After you signed, you will take part in the study (FGD or IDI). To get deep experiences of you, we separate male and female participants for FGD. The activity will meet once for interview will take about 30 min and FGD 60 min. If you have any concern, you have the right to ask your questions at any point. Also, you can choose not to take part in the study and if you join once, also you can choose to quit at any time. There is no penalty to you and we will not be offended. There will be no penalty for skipping questions or deciding you want to end your participation.

The primary benefit of participating in the study is that the finding will be used to further for program, policy development and implementation of the contextualized and sub-group based strategies. Additionally, to compensate your time the researchers will provide you 50 ETB (1.9 USD) per person. Participation in the study is voluntary and information that will be collected from you will assured confidentiality. For FGD participants: At the beginning of the focus group session, the group will create a mutually agreed upon code of conduct that covers issues of confidentiality and ethics within the group. The results will be shared with national stakeholders and we write scientific reports and articles for sharing of the findings.

If you have any concerns and worries you can call the PI of this research, cell phone or email.

Phone: +251960116581

Email: [ayanayoom@gmail.com](mailto:ayanayoom@gmail.com)

**Street children FGD and In-depth interview guide**

**Part I: Study participants’ biodata sheet**

1. Participants background: Age
2. Sex male Female
3. Where did you born?.............
4. Marital status: single informally married
5. Educational background: Never had been into school Primary (1 to 6) Junior
6. How many years you lived in the street? Newly joined one year 2-5 years

5+ years

1. Current schooling status: in school dropped

**part II: The study participants’ reasons that cause them to flee into the street and initiate the process of flee into the street.**

This part is an interview guide to explore your experiences the reasons that cause street children to flee into the street and initiate the process of flee into the street. To continue this part again we ask your permission for the tape recording. Again your responses are confidential in whatever and where ever.

- 1. Currently, are you into schooling? If yes, who are sponsoring you? If you are not into schooling why you dropped your schooling?
  2. **From your experiences, what are the reasons that cause street children to flee into the street and initiate the process of flee into the street?**

1. Pushing factors (*poverty, separation of parents, family death, economic decline, etc.)*
2. Pulling factors (*enticements* *of apparent freedom, financial independence, peer influence, adventure and city glamour)*
3. How do you initiate the process of flee into the street?
4. What happened to you in the first day of arrival to street from home?
5. Who support you on the first day of arrival to the street?
6. To support yourself, what actions you did in the first arrival?
7. Where do you spend over the night? Is there any separate place for girls and boys?
8. When you join this street life, how did you socialize with other street children? What measures have you taken to familiarize yourself with others? Please describe.

**2.2. Challenges of living in the street**

1. Looking back at your history, can you tell us about your health and lifestyle experiences of street life?
2. What are the major challenges of being a street child?
3. what types of plights, you experienced in the street?
4. Critical shortage of coverage of basic needs (Probing questions……. *shortage of food, cloth, education* fee, *shelter…...etc*.).
5. Fragmentation of social ties and networks (probing……*biological family, community)*
6. Harassments (probing ……*Physical, sexual and verbal…etc.*)
7. Child trafficking (in and out of the city)
8. Do you think that you are liable/ vulnerable for high risk sexual behaviors? Why? (Probing: among street children, outsider community, strangers, group sex, homo/ heterosexual, oral/anal sex etc. Please describe.
9. When you experienced the life difficulties, how you get out of it? (probing using stimulants like khat, marijuana, glue, hashish, benzene, local drinks etc.)

**Part III: Means of survival and coping mechanisms of street life plights**

This part is an interview guide to explore your experiences the means of survival and coping mechanisms of street life plights. To continue this part again we ask your permission for the tape recording. Again your responses are confidential in whatever and where ever.

- 1. What is/ are the major sources of your income to sustain your daily life? (Probing: survival sex, sex exchange, manual work, shoe shine etc.)
  2. How do your survival and cope with street life plights (group life, collective security etc.)?
- What are the advantage of forming a group?
- Who are responsible to make and lead the group?
- How you manage and governing the group members?
  1. From your experiences, do you think you are risk for criminal attack? If yes, how do you protect yourselves from any criminal attack? If yes, what are the causes for Inter or intra-group conflict? (probing*…. Break of group norm and culture, competition for boyfriends/girlfriend, rumor etc.).*
- If the conflict happens within or between groups, how you manage the conflict? *(Probing …… orientation, mediate disputes, nonviolent* *behavior).*
- In the conflict management what is the role of females?
- If the negotiation failed what will happen within the group or between the group? (Probing…*self-defense, group desperation*? Then what next?)
  1. Is there any support to the group to survive the plights of street?
     1. If yes, what you do? (probing*…. manual work, scavenging, begging, and engaging in paid sex)*
     2. Sharing vital information (probing …...*sharing vital information like free secure and popular places, market and commercial areas)*
     3. Is there individual based mandate within the group to survive? if yes, what type of mandate? (Probing ……*sharing information and resources*, *welcoming newcomers to the group and orienting survival skills and socializing with the group)*
  2. From your experience, do you participate in sex as a means of survival? If yes, who participate in the paid sex (males or female)? What do you do by the money you got from paid sex? (probing…. *multiple partners, your autonomous to have a sex, paid sex, willingness, heterosexual, homosexual, rape, gang rape,* *forced sex etc.)*

**Part IV: Available government, community and NGOs programs and initiatives to halt the plights of street children**

This part is an interview guide to explore your experiences available government and NGOs programs and initiatives to halt the plights of street children. To continue this part again we ask your permission for the tape recording. Again your responses are confidential in whatever and where ever.

**4.1.** So far, what are available government, communities and NGOs programs and initiatives to halt the plights of street children? **(Probing……...***legal protection, strategies and policies, government political attention …. etc.*)

**4.1. 1.** If there is no any programs and initiatives to halt your street life plights, what happened to you do to absence of these programs and initiatives?

4.2. To address your problems, how do you feel that the government can do in the future?

**Part V: Type of strategies and policies do you perceive and propose to government and NGOs, communities, and other interested stakeholders to alleviate and prevent street life plights**

This part is an interview guide to explore your experiences the type of strategies and policies do you perceive and propose to government and NGOs, communities, and other interested stakeholders to alleviate and prevent street life plights. To continue this part again we ask your permission for the tape recording. Again your responses are confidential in whatever and where ever.

- 1. What type of strategies and policies do you perceive and propose to government and NGOs, communities, and other interested stakeholders to alleviate and prevent street life plights**?** Probing …. *Separate shelter, health education, and life coaching access, child protection, self-help, community support, Reintegration etc.*
  2. Do you have anything you want to add/say more about your experiences?

Dear Participants

I thank you for your contribution!
