# Supplementary material for: Initiation into the street, challenges, means of survival and perceived strategies to prevent plights among street children in Addis Ababa, Ethiopia 2019: A phenomenological study design
Source: PLoS One. 2022 Aug 29;17(8):e0272411. doi: 10.1371/journal.pone.0272411 (PMC9423604; doi:10.1371/journal.pone.0272411)
Supplement: S2 File — (DOCX) [file pone.0272411.s002.docx]

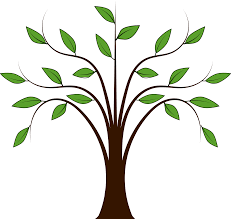
 Street children

**Health Policies** Programs and Political **will**

Health Policies

**Child trafficking**

Reintegration Social protection services Self-help

Community support Health Education

Coaching

Means of Survival Forming a group Sex as a means of survival

**Being Street Child**

**Challenges in the street Cloth Education fee**

**Lack of Basic Needs Food Child trafficking**

**Sexual Harassment Physical Harassments Verbal**

In group what we expect Abuse Neglect Abuse Alone

Initiation process

PUSHING FACTORS

PULLING FACTORS

*Fig.3: Cloud Tree*
