# Supplementary material for: Initiation into the street, challenges, means of survival and perceived strategies to prevent plights among street children in Addis Ababa, Ethiopia 2019: A phenomenological study design
Source: PLoS One. 2022 Aug 29;17(8):e0272411. doi: 10.1371/journal.pone.0272411 (PMC9423604; doi:10.1371/journal.pone.0272411)
Supplement: S3 File — (DOC) [file pone.0272411.s003.doc]

Separate shelter

Social network fragmentations

From biological families

From community

Food

Support for self-help

Perceived and proposed

Strategies to prevent and address plights

Cloths

The home of plights and challenges

Harassments

Shortage of basic needs

Shelter

Denial of social protections

Child trafficking

Education fee

Within city

Out of the city

Initiation process

Alone

Alone

By group

By group

Health education and coaching

Reintegration

Neglected

Poverty

sexual

Child abuse

Physical

City *glamour*

School dropout

Traditional values

Verbal

Family size

Peer influence

Adventure

Pushing factors

Economy problem

Ideal freedom

Pulling factors

Family size

Financial independence

ROOT CAUSE TO FLEE TO THE STREET

Land grape

Fig. Word Tree
